# Supplementary figures and images for: A method for remotely measuring physical function in large epidemiologic cohorts: Feasibility and validity of a video-guided sit-to-stand test
Source: PLoS One. 2021 Nov 19;16(11):e0260332. doi: 10.1371/journal.pone.0260332 (PMC8604329; doi:10.1371/journal.pone.0260332)

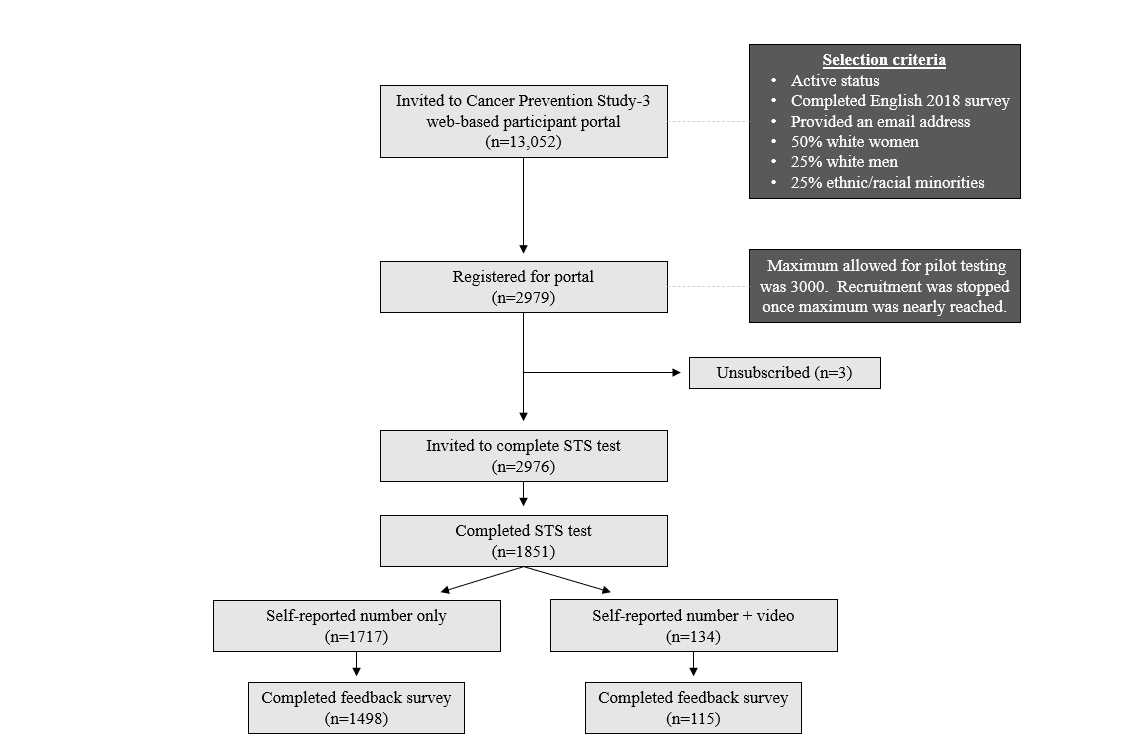


Supplemental Figure 1. Flow of Participants

Supplement: S1 Fig — (DOCX) [file pone.0260332.s001.docx]

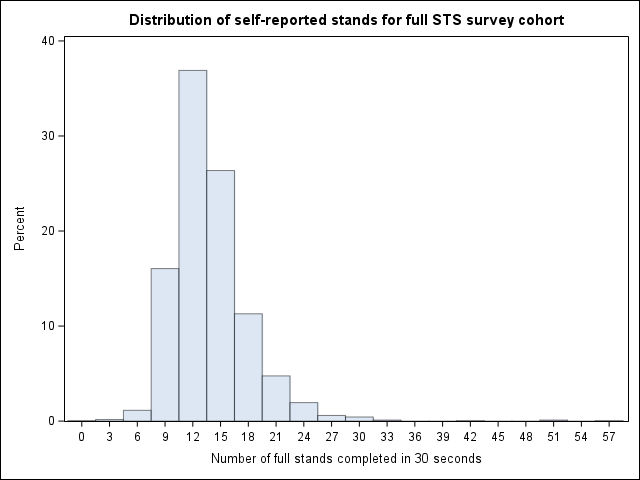


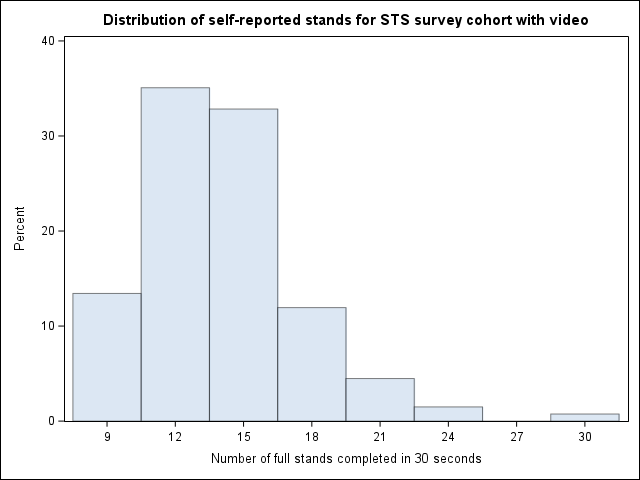

Supplement: S2 Fig — (DOCX) [file pone.0260332.s002.docx]
